# Extended Effectiveness of the Etonogestrel-Releasing Contraceptive Implant and the 20 µg Levonorgestrel-Releasing Intrauterine System for 2 Years Beyond U.S. Food and Drug Administration Product Labeling

**DOI:** 10.9745/GHSP-D-17-00296

**Published:** 2017-12-28

**Authors:** Moazzam Ali, Luis Bahamondes, Sihem Bent Landoulsi

**Affiliations:** a Department of Reproductive Health and Research, World Health Organization, Geneva, Switzerland.; b Family Planning Clinic, Department of Obstetrics and Gynaecology, Faculty of Medical Sciences, University of Campinas, Campinas, São Paulo, Brazil.

## Abstract

Recently published evidence from 2 large studies find that the duration of effectiveness of the etonorgestrel-releasing contraceptive implant to be at least 5 years (compared with the current 3-year label), and for the 20 µg levonorgestrel-releasing intrauterine system at least 7 years (compared with the current 5-year label).

## BACKGROUND

Contraceptive implants, the levonorgestrel-releasing intrauterine system (LNG IUS), and the copper-bearing intrauterine device (IUD) are long-acting reversible contraceptives (LARCs) with high contraceptive effectiveness. The cumulative pregnancy rates in the first 3 years of use of LARCs is 0.9 per 100 woman-years.^1^ In comparison, the percentages of women experiencing an unintended pregnancy during the first year of typical use of short-acting methods are much higher, including for male condoms (18%), the diaphragm (18%), Depo-Provera injectables (6%), and combined oral contraceptive pills or progestin-only pills (9%).^2^

The high effectiveness of LARCs is equal in women of all ages, whereas younger women using the pill, patch, or vaginal ring have a significant increase in contraceptive failure in comparison with failure rates among older women.^3^ Moreover, LARCs convey many other advantages for clients in terms of convenience, satisfaction, ease of continuation, likelihood of avoiding unintended/unwanted pregnancy, and noncontraceptive benefits.^3^^–^^8^ For these reasons, LARCs should also be among the readily available contraceptive choices for women, including young and nulliparous women. If their duration of effective use were to be extended, that would likely be another perceived benefit of LARCs.

LARCs should be among the readily available contraceptive choices for women.

## BRIEF DESCRIPTION OF HORMONAL LARCs

### Etonogestrel-Releasing Implant

The etonogestrel (ENG)-releasing implant contains 68 mg ENG embedded in 1 ethylene-vinyl-acetate rod^9^ (marketed in the United States as Implanon and Nexplanon, Merck & Co., Inc., Whitehouse Station, NJ, USA). ENG is the biologically active metabolite of desogestrel used in some combined and progestogen-only contraceptive pills. The ENG-releasing implant is currently labeled for 3 years of use. The original 1-rod ENG-releasing contraceptive implant had first regulatory approvals in 1998 in Indonesia.

**Mechanism of action.** Contraceptive implants act by binding to their receptors located in diverse target cells, which are distributed along the hypothalamic-pituitary-gonadal-genital tract axis. The implant has the ability to interfere with several key processes required for gamete encounter and fertilization. The progestins work both by suppressing and altering ovulation and by thickening the cervical mucus.^9^ They also restrict or suppress the access of fertile spermatozoa to the site of fertilization.

### Levonorgestrel-Releasing Intrauterine System

The LNG IUS is a T-shaped device that is labeled for up to 5 years of use. It has been available in Europe since 1990 and in the United States since 2000. It is marketed under the name Mirena (Bayer Schering Pharma, Berlin, Germany) and contains 52 mg levonorgestrel.^10^ The LNG IUS consists of a rate-controlling membrane, which releases 20 µg/day, that serves to regulate the rate of hormonal release.^11^

**Mechanism of action.** The contraceptive and therapeutic effects of the LNG IUS are mainly based on 3 local effects of LNG in the uterus: thickening of the cervical mucus, inhibition of sperm motility and function inside the uterus and the fallopian tubes, and prevention of fertilization and endometrial growth.^11^

## PHARMACOKINETIC DATA SUPPORT LONGER EFFICACY

### ENG-Releasing Implant

The ENG-releasing implant, with 68 mg of ENG as the active ingredient, releases, on average, 60–70 μg/day in weeks 5–6, decreasing to about 35–45 μg/day by the end of the first year, 30–40 μg/day by year 2, and then to 25–30 μg/day at the end of the third year.^12^ The bioavailability remains constant and close to 100%, and the elimination half-life of the parent compound is around 25 h.^13^ Existing data suggest that an ENG concentration of >90 picograms per milliliter (pg/mL) is necessary to effectively prevent ovulation.^14^ In normal-weight women (i.e., body mass index [BMI]=18.5–24.9 kg/m^2^), the average ENG concentrations at 2 and 3 years post-insertion are 194 and 156 pg/mL, respectively. Pharmacokinetic (PK) analysis shows that at the end of the labeled life span of the ENG-releasing implant (i.e., 3 years), the serum hormone levels are above the threshold for effective contraception,^13^^,^^15^ indicating that the ENG-releasing implant is likely to be effective for contraception up to the fourth and fifth years of use.^16^^–^^18^

Pharmacokinetic analysis shows that at the end of the labeled life span of the ENG-releasing implant, the serum hormone levels are above the threshold for effective contraception.

Moreover, McNicholas et al.^17^ reported that among ENG-releasing implant users with serum ENG results, the median ENG level was 207.7 pg/mL (range 63.8–802.6 pg/mL) at the end of the third year, 166.1 pg/mL (range 25.0–470.5 pg/mL) at the end of the fourth year, and 153.0 pg/mL (range 72.1–538.8 pg/mL) at the end of the fifth year. Thus, at the end of fifth year, the median ENG concentrations are above 90 pg/ml, which effectively prevents ovulation.^14^ So even if blood levels with the ENG-releasing implant dropped lower in still later years to the point where some ovulation were to occur, efficacy should in principle remain excellent for a time beyond 5 years. Nevertheless, some caution should be taken as there may be variation among women.

### LNG IUS

The LNG IUS has exceptionally good efficacy because it works by both a local effect of the hormone on cervical mucus and uterine milieu and a systemic effect to impair ovulation. Blood levels can be taken as indicative of both effects. During the first year of use, the LNG IUS releases 20 μg of LNG every 24 hours, declining slowly over the labeled lifetime of the device. Release of the hormone decreases to 11 μg per 24 hours by the end of 5 years, with an average release rate of 14 μg per day over the life of the device.^19^^,^^20^

A recent PK study showed that LNG plasma levels decline over time, with the greatest relative drop occurring between years 2–3 of use, followed by a sustained plateau from years 4–8.^21^ Women who used the LNG IUS for ≥6 years had statistically significantly lower but still similar LNG serum levels than women who used the LNG IUS ≤5 years (126±44 pg/mL vs.157±62 pg/mL, respectively; *P*=.01); however, there were no pregnancies reported in either group.^21^

A recent pharmacokinetic study showed that the greatest relative drop in LNG plasma levels among LNG IUS users occur between years 2–3 of use, followed by a sustained plateau from years 4–8.

## CLINICAL STUDIES ALSO SUPPORT LONGER EFFICACY

### Extended Efficacy of the ENG-Releasing Implant to 5 Years

Recently, a multicenter clinical trial conducted by the World Health Organization (WHO) compared the clinical performance and contraceptive efficacy of Jadelle and Implanon with a nonrandomized control group of women using the copper-bearing TCu380A IUD.^18^ The trial was originally designed for 3 years and was conducted in Brazil, Chile, the Dominican Republic, Hungary, Thailand, Turkey, and Zimbabwe. Women in the IUD group were matched by age (in 5-year bands) to every second woman allocated to an implant. At the 36-month visit or earlier, all study participants were invited to participate in an extended phase of the study for an additional 2 years. A subset of 390 ENG-releasing implant and 522 LNG-releasing implant participants consented to extended use up to 5 years. The main outcome of the extended study was to obtain the 4- and 5-year annual and cumulative effectiveness rates, continuation rate, and side effects for both contraceptive implant systems.

During the extended period through 5 years of use, while the products were in situ, no subdermal implant user became pregnant among the 7,060 and 10,883 woman-months of observation for the ENG-releasing and LNG-releasing subdermal implant group, respectively (Table 1). At the completion of 5 years, the cumulative pregnancy rates among ENG- and LNG-releasing implant users were statistically equivalent: 0.6 (95% confidence interval [CI], 0.2 to 1.8) and 0.8 (95% CI, 0.2 to 2.3), respectively. From the time of insertion to the extended phase of the study, ENG-releasing implant users accumulated more than 22,000 woman-months of use. During the same time frame, the 2-year pregnancy rate in the copper-bearing IUD group compared with the 2 implant groups combined was 4.1 per 100 woman-years (95% CI, 2.5 to 6.5).

**TABLE 1.** Pregnancy Data Among LNG- and ENG-Releasing Subdermal Implant Users Through 5 Years of Use From Ali et al., 2016^18^

	LNG-Releasing Implant				ENG-Releasing Implant			
	Years 1–3^a^	Year 4^a^	Year 5	Years 1–5 (Cumulative)	Years 1–3^a^	Year 4^a^	Year 5	Years 1–5 (Cumulative)
No. of women	997	470	330		995	311	204	
Woman-months of observation	28670	6,254	4,629	30,325	28786	4,606	2,454	22,044
No. of pregnancies	3^b^	0	0	3	3^b^	0	0	3
Cumulative pregnancy rates per 100 woman-years^c^ (95% CI)	0.4 (0.1–1.4)		0.8 (0.2, 2.3)		0.4 (0.1–1.4)			0.6 (0.2, 1.8)

Abbreviations: CI, confidence interval; ENG, etonogestrel; LNG, levonorgestrel.

a Cut-off for 3 years was at 38 months post-insertion while year-4 data started at 36 months post-insertion, resulting in a 2-month overlap in data. Woman-months of observation between these 2 time periods, however, is not additive.

b Pregnancy data from the first 3 years reported in Bahamondes et al., 2015.^22^

c Kaplain-Meier rates.

A recent multicenter WHO clinical trial found no pregnancies among implant users through 5 years of use.

Moreover, recently, McNicholas et al. reported results of a large follow-up study of the ENG-releasing implant and the LNG IUS.^17^ For the ENG-releasing implant, 223 users who continued for more than 12 additional months beyond the labeled life span had no pregnancies per 100 woman-years (1-sided 97.5% CI, 0 to 1.48) at the fourth year of use, and 102 participants who continued for more than 24 additional months also had zero pregnancies per 100 woman-years (1-sided 97.5% CI, 0 to 2.65) at 5 years (Table 2).

**TABLE 2.** Pregnancy Data Among ENG-Releasing Implant Users Through 5 Years From McNicholas et al., 2017^17^

	Year 4	Year 5
**No. of women**	223	102
**Woman-years of observation**	444.0	
**No. of pregnancies**	0	0
**Pregnancy rate per 100 woman-years (1-sided 97.5% CI)**	0 (0, 1.48)	0 (0, 2.65)

Abbreviations: CI, confidence interval; ENG, etonogestrel.

### Extended Efficacy of the LNG IUS to 7 Years

Results of a WHO-sponsored, open-label, 7-year randomized controlled trial were recently published from 20 centers, 11 of which were in China.^23^ The main objectives were to compare rates of unintended pregnancy, method continuation, and reasons for removal among women using the 52-mg LNG IUS (daily release 20 µg) or the TCu380A IUD. Over the 7-year period, 7 pregnancies occurred among LNG IUS users, all intrauterine pregnancies. The cumulative 7-year pregnancy rate of the LNG IUS was 0.5 per 100 woman-years (95% CI, 0.3 to 0.8; standard error 0.2) (Table 3). No pregnancy occurred from 8 to 11 years of use in either the 1,342 woman-years of observation of the TCu380A or the 681 woman-years of observation of the LNG IUS, based on 682 TCu380A IUD users and 398 LNG IUS users starting the eighth year of use. The study data concludes that the 52-mg LNG IUS is safe with very high contraceptive efficacy and very low cumulative pregnancy rates through 7 years of use.^23^

**TABLE 3.** Comparative Efficacy of the TCu380A IUD and the 52-mg LNG IUS Over 7 Years From Rowe et al., 2016^23^

	TCu380A IUD	LNG IUS
No. of women	1,871	1,884
Woman-years of observation	10,088	7,903
No. of pregnancies	33	7^a^
Cumulative pregnancy rate per 100 woman-years (95% CI)	2.5 (2.1, 2.9)	0.5 (0.3, 0.8)

Abbreviations: CI, confidence interval; IUD, intrauterine device; LNG IUS, levonorgestrel-releasing intrauterine system.

a No pregnancies were reported in years 6 and 7.

In a multicenter WHO randomized controlled trial, the cumulative 7-year pregnancy rate of the LNG IUS was 0.5 per 100 woman-years.

Supporting the findings of the WHO study,^23^ McNicholas et al.^17^ also reported the effectiveness of the 52-mg LNG IUS into the sixth and seventh year. Among the 496 women using this LNG IUS, 696.9 woman-years of follow-up were completed, with only 2 total pregnancies reported in the sixth and seventh year (Table 4). The failure rate in the sixth year of use of the 52-mg LNG IUS is calculated as 0.25 per 100 woman-years (95% CI, 0.04 to 1.42), and in the seventh year, 0.43 per 100 woman-years (95% CI, 0.08 to 2.39). These failure rates are comparable with the published failure rate of the device's current U.S. Food and Drug Administration (FDA)-labeled period of 5 years. The study concluded that the LNG IUS continues to be highly effective for at least 2 years of additional use beyond its labeled life span.

**TABLE 4.** Pregnancy Data Among Users of the 52-mg LNG IUS From McNicholas et al., 2017^17^

	Year 6	Year 7
**No. of women**	496	
**Woman-years of observation**	696.9	
**No. of pregnancies**	2	
**Pregnancy rate per 100 woman-years (95% CI)**	0.25 (0.04, 1.42)	0.43 (0.08, 2.39)

Abbreviations: CI, confidence interval; LNG IUS, levonorgestrel-releasing intrauterine system.

Studies from the early development of the LNG IUS also found no pregnancies in years 5 to 7, further supporting the longer duration of efficacy.^24^^–^^28^

## IMPLICATIONS

Highly effective LARCs can be an excellent contraceptive choice for clients wishing to avoid unplanned pregnancies. Recent studies find that both the ENG-releasing contraceptive implant and the 20 µg/day LNG IUS are highly effective for at least an additional 2 years beyond their FDA labels—from the current 3-year label for ENG-releasing implants to at least 5 years, and from the current 5-year label for the LNG IUS to at least 7 years—and with far better efficacy than many other contraceptive methods.

Recent studies find that both the ENG-releasing implant and the 20 µg LNG IUS are highly effective for at least an additional 2 years beyond their FDA labels.

Extending the labeled duration of effective use for ENG-releasing subdermal implants and the LNG IUS would have many benefits for women and for family planning programming. Access to choice of contraceptive methods is considered a basic right for women and couples,^29^ and extending use of these methods could help with access and choice for women when considering contraceptive methods. Longer duration is safer for users, requires less frequent removal and insertion cycles, and reduces the chances of procedural errors. Also, extended use saves the client time and money, and may be cost effective for the health system. For example, international donor agencies currently pay US$9 per unit for an ENG-releasing implant; if 2 additional years were added to its life span, the commodity cost per couple-year of protection would drop from US$3 to US$1.80.^18^

In interpreting these studies, a few limitations should be taken into account including the observational nature of one of the studies,^18^ loss to follow-up, and limited data on women with a body mass index (BMI) >30 kg/m.^2^^,^^18^

The manufacturers of these products should take note of the findings of these studies and seriously consider relabeling their duration of use. In the current situation, it is unclear whether the licensed owners of these products will be interested in taking steps toward this change. Given the major advantages of these methods and the benefits to women to continue using a method they are already successfully using, programs, policy makers, and providers should take note of these findings and provide women using these methods the option, should they wish to continue their use for an additional 2 years. It is a matter of informed choice. A systematic review summarizing the safety and effectiveness of extended use of these LARCs would be an important step in making recommendations for WHO's medical eligibility criteria for extended use.

## Notes

**Peer Reviewed**

**First Published Online:** December 20, 2017

**Funding:** This work was funded by the UNDP-UNFPA-UNICEF-WHO-World Bank Special Programme of Research, Development and Research Training in Human Reproduction (HRP), a cosponsored program executed by the World Health Organization (WHO).
